# Postoperative changes in the pharyngeal airway space through computed tomography evaluation after mandibular setback surgery in skeletal class III patients: 1-year follow-up

**DOI:** 10.1186/s40902-021-00319-1

**Published:** 2021-08-27

**Authors:** No Eul Kang, Dae Hun Lee, Ja In Seo, Jeong Keun Lee, Seung Il Song

**Affiliations:** Department of Oral and Maxillofacial Surgery, Institute of Oral Health Science, Ajou University School of Medicine, 164, Worldcup-ro, Yengto-gu, Suwon-si, Gyeonggi-do 16499 Republic of Korea

**Keywords:** Mandibular setback surgery, Bimaxillary surgery, Class III malocclusion, Pharyngeal airway, CBCT

## Abstract

**Background:**

This study evaluated the pharyngeal airway space changes up to 1 year after bilateral sagittal split osteotomy mandibular setback surgery and bimaxillary surgery with maxillary posterior impaction through three-dimensional computed tomography analysis.

**Methods:**

A total of 37 patients diagnosed with skeletal class III malocclusion underwent bilateral sagittal split osteotomy setback surgery only (group 1, *n* = 23) or bimaxillary surgery with posterior impaction (group 2, *n* = 14). Cone-beam computed tomography scans were taken before surgery (T0), 2 months after surgery (T1), 6 months after surgery (T2), and 1 year after surgery (T3). The nasopharynx (Nph), oropharynx (Oph), hypopharynx (Hph) volume, and anteroposterior distance were measured through the InVivo Dental Application version 5.

**Results:**

In group 1, Oph AP, Oph volume, Hph volume, and whole pharynx volume were significantly decreased after the surgery (T1) and maintained. In group 2, Oph volume and whole pharynx volume were decreased (T2) and relapsed at 1 year postoperatively (T3).

**Conclusion:**

In class III malocclusion patients, mandibular setback surgery only showed a greater reduction in pharyngeal airway than bimaxillary surgery at 1 year postoperatively, and bimaxillary surgery was more stable in terms of airway. Therefore, it is important to evaluate the airway before surgery and include it in the surgical plan.

## Background

Many patients with skeletal class III malocclusion have esthetic and functional problems, and orthognathic surgery accompanying bilateral sagittal split osteotomy (BSSRO) setback surgery is administered to improve them. This surgery provides satisfactory results to patients by improving the mastication, pronunciation, esthetics, and psychological factors of the patient by changing the skeletal position of the mandible alone or both mandible and maxilla.

Orthognathic surgery affects not only hard tissue but also soft tissue and affects the stomatognathic system overall. The oropharyngeal complex is composed of the hyoid bone and the muscles connected to it, and is anatomically and functionally related to the mandible, resulting in changes after orthognathic surgery with mandible movement. When the mandible is moved backward by surgery, the tongue, hyoid bone, and muscles attached are located posterior to its original position, and as a result, the airway volume is narrowed [1–8]. Many previous studies have shown that mandible setback surgery narrows the pharyngeal airway [4, 9–11]. Several studies have reported that postoperative obstructive sleep apnea (OSA) occurred as a result [4, 6, 9, 11, 12]. On the other hand, in the case of BSSRO setback surgery with Le Fort I osteotomy, there are reports that the decrease in pharyngeal airway volume is less than in patients who underwent mandibular setback surgery only, and there are some studies that the anteroposterior (AP) dimension is rather increased in the upper pharyngeal airway after the surgery [13–15].

In the previous study, two-dimensional analysis using cephalometric radiographs was performed to investigate the change of pharyngeal airway, but there were limitations in the analysis of the three-dimensional (3D) pharyngeal airway space or volume [16, 17]. Cone-beam computed tomography (CBCT) is a 3D image that is very useful for preoperative diagnosis, operation planning, and evaluation of postoperative results. It also provides high quality while reducing scanning time and irradiation compared to conventional computed tomography (CT). In addition, a CT image is useful for evaluating pharyngeal airway including both hard tissue and soft tissue, and it is possible to visualize a 3D image to help intuitive understanding. Nowadays, as many CT scans and programs are developed, there are some studies evaluating the volumetric evaluation of the airway after surgery [12, 18–22]. Most of them were limited to only BSSRO setback surgery, and there were not many cases that the volume change was observed regularly until 1 year.

From our experience, airway narrowing was seen on the radiograph in some patients after mandibular setback surgery and some patients complained of airway-related symptoms such as snoring immediately after the surgery. Over time, these symptoms subsided and it was expected that there would be changes in the airway over time. In particular, in the case of bimaxillary surgery with posterior impaction, the change in the airway was expected to show a different pattern because the mandible was not simply positioned posteriorly but the maxilla was rotated clockwise. Accordingly, we hypothesized that the three-dimensional volume and anteroposterior distance of the airway would be different over time depending on the surgical methods.

The purpose of our study was to evaluate the pharyngeal airway space changes up to 1 year after BSSRO surgery with or without Le Fort I osteotomy through 3D CT analysis.

## Materials and methods

### Patients

From February 2014 to April 2019, 37 patients (19 men, 18 women; mean age 23.2 ± 5.47 years) diagnosed with skeletal class III malocclusion and who underwent BSSRO setback surgery or bimaxillary surgery by the same operator at the Ajou University School of Medicine (Suwon, Korea) were included in this study (Table 1). All the patients were followed for more than 1 year and pre- and postorthodontic treatments were provided. Patients who had craniofacial syndromes, such as cleft lip and palate, cranial trauma, and upper respiratory lesions, or who underwent other surgery such as genioplasty were excluded.

**Table 1:** Surgical movement of group 1 and group 2

Surgical variable	Mean ± SD		***P*** value
	Group1 (***n*** = 23)	Group2 (***n*** = 14)	
Maxillary posterior impaction (mm)^a^	–	4.23 ± 3.14	–
Advancement of maxilla (mm)^b^	–	0.81 ± 2.11	–
Mandibular setback amount (mm)^c^	4.85 ± 4.34	6.81 ± 5.14	0.223
Changes of mandibular plane angle (°)^d^	3.54 ± 4.56	2.17 ± 4.24	0.371

^a^Maxillary posterior impaction was defined as the upward movement of the posterior nasal spine

^b^Advance of maxilla was defined as the forward movement of the anterior nasal spine

^c^Mandibular setback amount was measured through the distance difference from sella vertical plane to B point

^d^Mandibular plane angle, the angle between FH plane and mandibular plane

Independent *t*-test was performed and *P* values < 0.05 are statistically significant

Patients were divided into two groups: (1) 1 jaw surgery patients (BSSRO setback surgery only), (2) 2 jaw surgery patients (Le Fort I osteotomy with posterior impaction and BSSRO setback surgery). The surgical method was different for group 1 and group 2. In the case of group 1, BSSRO setback surgery was performed, followed by rigid fixation. In group 2, after Le Fort I osteotomy, the maxilla was moved to the desired position, rigid fixation was performed, and mandible BSSRO setback surgery was performed as in group 1. After surgery, all patients had to wear wafers for 2–4 weeks to stabilize occlusion, and postorthodontic treatment was performed afterwards. Table 1 shows the amount of surgical movement in each group.

Ajou University School of Medicine Institutional Review Board approved the study protocol (Approval No. AJIRB-MED-MDB-20-078).

### CBCT examination and measurements

CBCT scans were taken before surgery (T0), 2 months after surgery (T1), 6 months after surgery (T2), and 1 year after surgery (T3). During a CT scan, in order to have a reproducible posture, patients were asked to take a natural head position. Patients were asked to stand and stare at the front mirror, and the lips, chin, and masticatory muscles were relaxed to take a reproducible posture.

CBCT (Dinnova3; HDX, Seoul, Korea) of Ajou University Dental Hospital was used, and the imaging conditions were 80 kVp, 7.0 mA, and scan time for 20 s. The slice thickness was 0.3 mm and the distance between slices was 1.0 mm. CT digital image files were exported using the Digital Imaging and Communications in Medicine (DICOM) format and imported into the InVivo Dental Application version 5 (Anatomage, San Jose, CA, USA). The landmarks, reference planes, and measurement points used in this study are as follows (Tables 2 and 3). Since the measured value varies according to the head position, re-orientation was performed by setting the nasion as the origin point, the Frankfort horizontal plane passing through the orbitale and the porion as the horizontal reference plane, and the sella vertical plane passing through the sella and basion as the sagittal reference plane.

**Table 2:** The landmarks and the reference plane

Landmark (abbreviation)	Definition
Landmarks	
Na (nasion)	“V” notch of frontal and nasal bones
Ba (basion)	Most inferior point of the occipital bone
Or (orbitale)	Most inferior point of the orbital contour
Po (porion)	Most superior point of the external auditory meatus
Se (sella)	Center of sella turcica
Anterior nasal spine (ANS)	Tip of the anterior nasal spine
B point (B)	Deepest point between pogonion and lower incisal alveolus
CV_1_	Most anterior inferior point of the anterior arch of the atlas
CV_2_	Most anterior inferior point of the body of the 2nd cervical vertebra
CV_4_	Most anterior inferior point of the body of the 4th cervical vertebra
Reference plane	
Frankfort horizontal (FH) plane	Faces through the right porion and both sides of the orbitalia
Midsagittal plane	A plane perpendicular to the FH plane passing through Na and Ba
Sella vertical (SV) plane	A plane perpendicular to the FH plane passing through Se, Ba, and Na
CV_1_ plane	A plane parallel to the FH plane passing through CV_1_
CV_2_ plane	A plane parallel to the FH plane passing through CV_2_
CV_4_ plane	A plane parallel to the FH plane passing through CV_4_

**Table 3:** Pharyngeal airway measurements

Measurements	Definition
Nph AP	Nasopharyngeal airway distance, anteroposterior distance at CV_1_ level
Oph AP	Oropharyngeal airway distance, anteroposterior distance at CV_2_ level
Hph AP	Hypopharyngeal airway distance, anteroposterior distance at CV_4_ level
Nph vol	Nasopharyngeal airway volume, top of the pharyngeal airway to CV_1_ plane
Oph vol	Oropharyngeal airway volume, between the CV_1_ plane and CV_2_ plane
Hph vol	Hypopharyngeal airway volume, from CV_2_ plane to CV_4_ plane
Whole pharynx volume	Sum of the Nph volume, Oph volume, and Hph volume
SV-B	The distance from the sella vertical plane to the B point

**Fig. 1:**
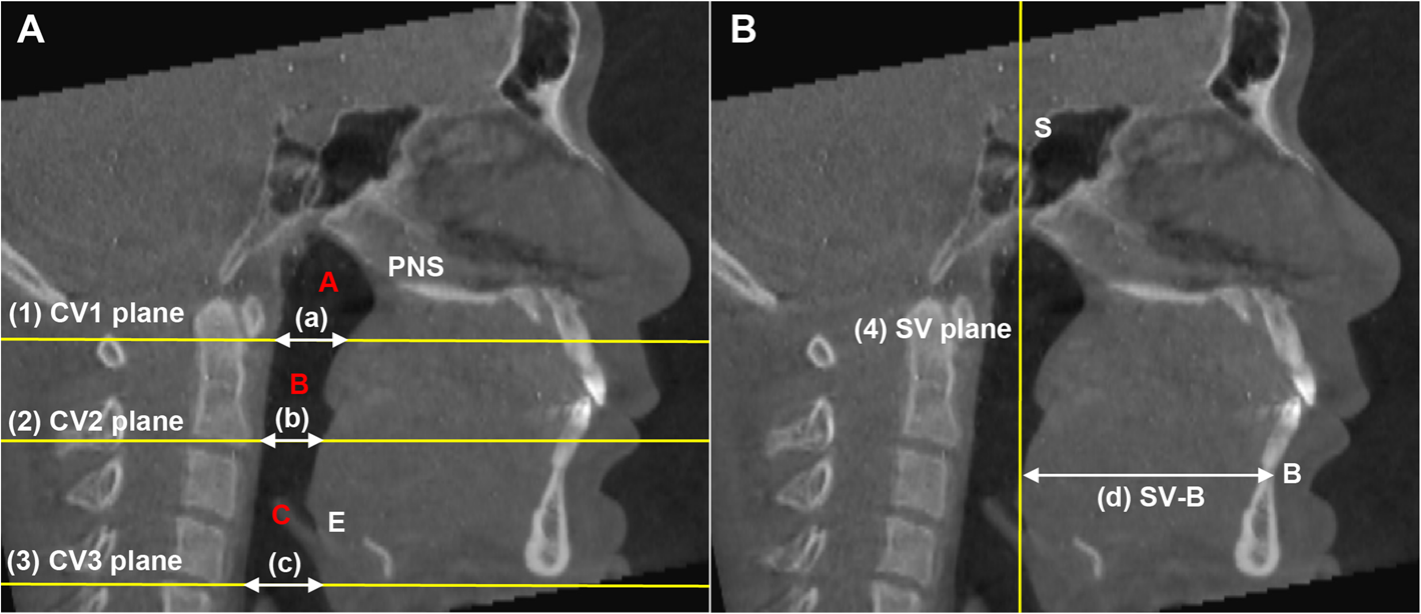
Pharyngeal airway division and measurements. **a** The airway space divided into three areas based on reference planes. (1) a plane parallel to the FH plane passing through CV_1_; (2) a plane parallel to the FH plane passing through CV_2_; (3) a plane parallel to the FH plane passing through CV_4_; A, the nasopharyngeal volume; B, oropharyngeal volume; C, hypopharyngeal volume; (a) nasopharyngeal antero-posterior distance; (b) oropharyngeal antero-posterior distance; (c) hypopharyngeal anteroposterior distance. **b** Measurement of SV-B. (4) sella vertical plane; (d) the distance from the sella vertical plane to the B point. *PNS* posterior nasal spine

**Fig. 2:**
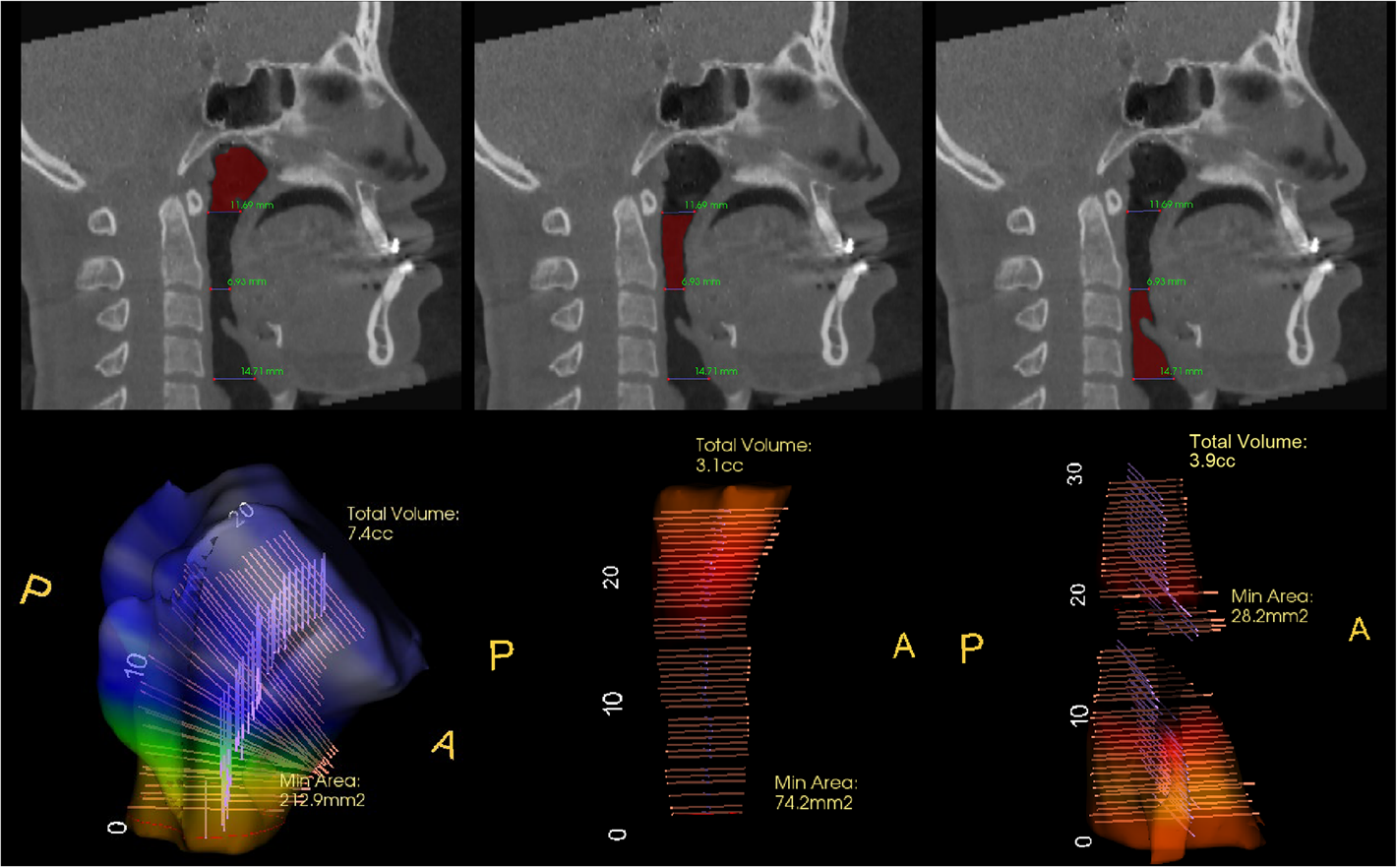
Pharyngeal airway volume measurement. Volume was measured through the InVivo Dental Application version 5

The upper airway space is surrounded by soft tissue and can be changed with surgery, dividing the upper airway space based on the plane passing through the cervical vertebra, CV_1_, CV_2_, and CV_4_ (Table 2). The airway space from the top of the pharynx to the CV_4_ plane is defined as the whole pharynx, and the nasal cavity and oral cavity are excluded. The pharynx was divided into subdivisions based on the CV_1_ plane and the CV_2_ plane, and defined as nasopharynx (Nph), oropharynx (Oph), and hypopharynx (Hph) from above (Table 3, Fig. 1). It was measured through the InVivo Dental Application version 5 (Fig. 2).

### Statistical analysis

Methodological errors in the measurement were minimized by double recording of the same investigator. The Shapiro-Wilk test was performed for the normalization test. A paired *t*-test was used to evaluate postoperative changes of pharyngeal space and relapse in each group. An independent *t*-test was used to determine the difference between groups. Probability values less than 0.05 were deemed statistically significant. SPSS Statistics version 23.0 software (SPSS Inc., Chicago, IL, USA) was used for all statistical analyses.

## Results

The measurements and volumes before surgery (T0), 2 months after surgery (T1), 6 months after surgery (T2), and 1 year after surgery (T3) in group 1 are shown in Table 4. Data for group 2 are shown in Table 5 and comparisons between groups are shown in Table 6. The mean of the mandibular setback amount was 4.85 ± 4.34 mm in group 1 and 6.81 ± 5.14 mm in group 2. The mean change of the mandibular plane angle was 3.54 ± 4.56° in group 1 and 2.17 ± 4.24° in group 2. Maxillary posterior impaction was done in group 2 with 4.23 ± 3.14 mm posterior nasal spine upward and 0.81 ± 2.11 mm advance of the anterior nasal spine (Table 1).

**Table 4:** Measurements and comparison of the pharyngeal airway in group 1 (BSSRO)

Variable	T0	T1	T2	T3	T0–T1	T0–T2	T0–T3	T1–T2	T2–T3
	Mean ± SD								
Nph AP	13.35 ± 2.80	12.39 ± 2.63	12.60 ± 2.70	12.48 ± 2.16	0.022*	0.070	0.065	0.630	0.761
Oph AP	12.30 ± 4.31	10.05 ± 4.20	10.20 ± 3.72	11.12 ± 4.33	0.000^**^	0.001^**^	0.025*	0.783	0.095
Hph AP	17.70 ± 4.55	15.93 ± 4.00	15.30 ± 4.14	16.67 ± 4.98	0.016*	0.003^**^	0.184	0.279	0.038*
Nph vol	6.23 ± 2.84	5.89 ± 2.93	5.65 ± 2.53	5.53 ± 2.73	0.239	0.049*	0.089	0.308	0.645
Oph vol	6.04 ± 2.80	4.24 ± 2.27	4.46 ± 2.49	4.10 ± 2.06	0.000^**^	0.000^**^	0.000^**^	0.516	0.245
Hph vol	7.80 ± 3.99	5.94 ± 3.84	5.93 ± 3.09	6.04 ± 3.65	0.000^**^	0.000^**^	0.002^**^	0.982	0.750
Whole pharynx vol	20.07 ± 8.22	16.07 ± 7.52	16.03 ± 6.70	15.67 ± 7.17	0.000^**^	0.000^**^	0.001^**^	0.962	0.622
SV-B	64.97 ± 7.88	60.12 ± 5.46	60.52 ± 6.09	60.86 ± 5.51	0.000^**^	0.000^**^	0.000^**^	0.466	0.321

^*^Significant at *p* < 0.05, ^**^Significant at *p* < 0.01

*BSSRO* bilateral sagittal split ramus osteotomy, *T0* before surgery, *T1* 2 months after surgery, *T2* 6 months after surgery, *T3* 1 year after surgery, *Nph AP* anteroposterior distance of nasopharynx, *Oph AP* anteroposterior distance of oropharynx, *Hph AP* anteroposterior distance of hypopharynx, *Nph vol* nasopharynx volume, *Oph vol* oropharynx volume, *Hph vol* hypopharynx volume, *SV-B* distance from the sella vertical plane to the B point

**Table 5:** Measurements and comparison of the pharyngeal airway in group 2 (BSSRO + Le Fort I osteotomy)

Variable	T0	T1	T2	T3	T0–T1	T0–T2	T0–T3	T1–T2	T2–T3
	Mean ± SD								
Nph AP	13.24 ± 2.87	12.79 ± 2.72	12.27 ± 2.96	12.58 ± 3.39	0.366	0.038*	0.173	0.312	0.644
Oph AP	14.52 ± 4.35	13.07 ± 5.53	12.11 ± 3.36	15.72 ± 9.75	0.427	0.076	0.614	0.377	0.196
Hph AP	18.00 ± 2.83	18.82 ± 3.76	18.69 ± 3.25	18.40 ± 3.48	0.367	0.447	0.634	0.838	0.619
Nph vol	7.43 ± 4.81	6.59 ± 4.00	6.72 ± 4.40	7.76 ± 4.30	0.185	0.129	0.525	0.762	0.009^**^
Oph vol	7.39 ± 4.00	6.14 ± 3.29	5.26 ± 2.57	7.11 ± 3.86	0.126	0.005*	0.328	0.064	0.010*
Hph vol	8.32 ± 3.75	8.53 ± 3.81	7.21 ± 2.91	7.94 ± 2.69	0.762	0.198	0.527	0.098	0.147
Whole pharynx vol	23.14 ± 10.78	21.25 ± 9.02	19.19 ± 7.68	22.81 ± 9.17	0.287	0.030*	0.721	0.083	0.007^**^
SV-B	63.93 ± 7.88	57.13 ± 7.45	57.35 ± 6.40	57.51 ± 6.60	0.000^**^	0.000^**^	0.000^**^	0.740	0.621

^*^Significant at *p* < 0.05, ^**^Significant at *p* < 0.01

*BSSRO* bilateral sagittal split ramus osteotomy, *T0* before surgery, *T1* 2 months after surgery, *T2* 6 months after surgery, *T3* 1 year after surgery, *Nph AP* anteroposterior distance of nasopharynx, *Oph AP* anteroposterior distance of oropharynx, *Hph AP* anteroposterior distance of hypopharynx, *Nph vol* nasopharynx volume, *Oph vol* oropharynx volume, *Hph vol* hypopharynx volume, *SV-B* distance from the sella vertical plane to the B point

**Table 6:** Comparison of the amount of changes between group 1 (BSSRO) and group 2 (BSSRO + Le Fort I osteotomy)

Variable		T0–T1		T1–T2		T2–T3		T0–T3	
		Average of change	***P*** value	Average of change	***P*** value	Average of change	***P*** value	Average of change	***P*** value
ΔNph AP (mm)	Group 1	0.96	0.422	− 0.21	0.284	0.12	0.549	0.87	0.756
	Group 2	0.45		0.51		− 0.31		0.66	
ΔOph Ap (mm)	Group 1	2.25	0.668	− 0.15	0.306	− 0.92	0.221	1.18	0.333
	Group 2	1.44		0.96		− 3.61		− 1.20	
ΔHph AP (mm)	Group 1	1.76	0.025^*^	0.63	0.568	− 1.37	0.078	1.03	0.225
	Group 2	− 0.82		0.14		0.30		− 0.40	
ΔNph vol (cm^3^)	Group 1	0.33	0.392	0.24	0.409	0.11	0.008^**^	0.69	0.116
	Group 2	0.84		− 0.14		− 1.04		− 0.33	
ΔOph vol (cm^3^)	Group 1	1.80	0.500	− 0.21	0.049^*^	0.37	0.004^**^	1.95	0.003^**^
	Group 2	1.26		0.88		− 1.85		0.29	
ΔHph vol (cm^3^)	Group 1	1.86	0.011^*^	0.01	0.094	− 0.11	0.293	1.76	0.093
	Group 2	− 0.21		1.32		− 0.74		0.38	
ΔWhole pharynx volume (cm^3^)	Group 1	3.99	0.246	0.01	0.141	0.37	0.004^**^	4.40	0.023^*^
	Group 2	1.89		2.06		− 3.62		0.34	

Independent *t*-test was performed

^*^Significant at *p* < 0.05, ^**^Significant at *p* < 0.01

*BSSRO* bilateral sagittal split ramus osteotomy, *T0* before surgery, *T1* 2 months after surgery, *T2* 6 months after surgery, *T3* 1 year after surgery, *Nph AP* anteroposterior distance of nasopharynx, *Oph AP* anteroposterior distance of oropharynx, *Hph AP* anteroposterior distance of hypopharynx, *Nph vol* nasopharynx volume, *Oph vol* oropharynx volume, *Hph vol* hypopharynx volume

### Nasopharyngeal airway change

In group 1, the average Nph AP was 13.35 mm preoperatively, and significantly decreased by 0.96 to 12.39 mm at 2 months after surgery (*p* < 0.05). It gradually increased and relapsed to 12.48 mm at 1 year after surgery (T3) without difference from T0 (*p* > 0.05). The mean preoperative Nph volume (Nph vol) was measured to be 6.23 cm^3^ and was 5.53 cm^3^ at 1 year postoperatively, with no difference before and after surgery (*p* > 0.05) (Table 4).

In group 2, the Nph AP was 13.24 mm, which was similar to the preoperative average of group 1, and 12.58 mm at 1 year after surgery (T3) without difference from T0 (*p* > 0.05). In group 2, the Nph vol was 7.43 cm^3^ before surgery (T0), and no significant change was observed until 6 months after surgery (*p* > 0.05). Nph vol significantly increased during the T2–T3 period (*p* < 0.01) and relapsed to 7.76 cm^3^ at 6 months postoperatively (T0–T3, *p* > 0.05). (Table 5).

### Oropharyngeal airway change

In group 1, the average preoperative measurement of Oph AP was 12.30 mm and decreased to an average of 10.05 mm at 2 months after surgery (*p* < 0.01), and remained reduced to 10.20 mm at 6 months after surgery (*p* < 0.01). After that, it increased slightly, and after one year, it became 11.12 mm, which is 1.18 mm lower than before the operation (*p* < 0.05). Oph vol decreased significantly from 6.04 cm^3^ preoperatively to 4.24 cm^3^ at 2 months postoperatively (*p* < 0.01) and remained decreased to 4.10 cm^3^ at 1 year postoperatively (*p* < 0.01) (Table 4).

In group 2, the preoperative mean of Oph AP was 14.52 mm, and no significant change was observed after surgery (*p* > 0.05). Oph vol decreased from an average of 7.39 cm^3^ preoperatively to 5.26 cm^3^ at 6 months postoperatively and then returned to 7.11 cm^3^ at 1 year postoperatively (Table 5).

Group 1 and group 2 were significantly different in the Oph vol change at the T1–T2, T2–T3, and T0–T3 periods (Table 6).

### Hypopharyngeal airway change

In group 1, the Hph AP value decreased significantly from 17.70 mm preoperatively to 15.93 mm at 2 months postoperatively and 15.30 mm at 6 months postoperatively (*p* < 0.05). Then Hph AP significantly increased at T2–T3 periods to 16.67mm (*p* < 0.05) and relapsed to preoperative value (T0–T3, *p* > 0.05). Hph vol significantly decreased from 7.80 cm^3^ preoperatively to 5.94 cm^3^ at 2 months postoperatively (*p* < 0.01) and remained decreased to 5.93 cm^3^ at 6 months and 6.04 cm^3^ at 1 year postoperatively (*p* < 0.01) (Table 4).

In group 2, Hph AP was 18.00 mm preoperatively and no change was observed until 1 year postoperatively (*p* > 0.05). The average Hph vol value was 8.32 cm^3^ preoperatively, and no significant change was observed until the first year (*p* > 0.05) (Table 5).

Group 1 and group 2 were significantly different in the Hph vol change at the T0–T1 stage (*p* < 0.05) (Table 6).

### Whole pharynx volume change

Whole pharynx volume is the sum of the nasopharyngeal, oropharyngeal, and hypopharyngeal airway volume values. Whole pharynx volume in group 1 significantly decreased from 20.07 cm^3^ preoperatively to 16.07 cm^3^ at 2 months postoperatively by 3.99 cm^3^ (*p* < 0.01) and decreased the most to 15.67 cm^3^ at 1 year postoperatively (*p* < 0.01) (Table 4).

In group 2, whole pharynx volume decreased from 23.14 cm^3^ before surgery to 21.25 cm^3^ at 2 months after surgery, but there was no significant change in comparison (*p* > 0.05). In addition, when comparing whole pharynx volume at T2 with T3, the average increased significantly from 19.19 cm^3^ to 22.81 cm^3^ (*p* < 0.01) and relapsed to a similar value to the preoperative average of 23.14 cm^3^ (T0–T3, *p* > 0.05) (Table 5).

Group 1 and group 2 were significantly different in the T2–T3 and T0–T3 stages (*p* < 0.05) (Table 6)

### B point change (setback amount)

SV-B is the distance from the sella vertical plane to the B point, which is measured to know the setback amount and relapse tendency. The difference between the SV-B values before and after surgery means the setback amount.

**Fig. 3:**
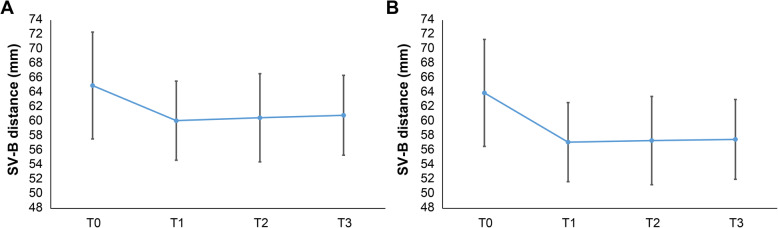
SV-B distance change. **a** Group 1 (bilateral sagittal split ramus osteotomy [BSSRO]). **b** Group 2 (BSSRO + Le Fort I osteotomy). *T0* before surgery, *T1* 2 months after surgery, *T2* 6 months after surgery, *T3* 1 year after surgery, *SV-B* the distance from the sella vertical plane to the B point

In group 1, the average preoperative SV-B was 64.97 mm, and in group 2, the average preoperative SV-B was 63.93 mm. The amount of change in SV-B value before and at 2 months after surgery was 4.85 mm in group 1 and 6.81 mm in group 2. In both groups, the SV-B value decreased significantly at T1 (*p* < 0.01), and the decreased value was maintained until T3 (Tables 4 and 5, Fig. 3).

## Discussion

BSSRO setback surgery and bimaxillary surgery are often used to treat class III malocclusion patients. Since there is a possibility of OSA after surgery, it is necessary to evaluate the pharyngeal airway before and after surgery. Because of its relatively simple and inexpensive advantage, many studies have used lateral cephalograms for preoperative and postoperative airway evaluation.

Riley and Powell [23] said that both CT and cephalometry are quite accurate for measuring the airway volume. However, Lenza et al. [24] argued that the upper airway could not be represented accurately by linear measurements only on cephalogram. Because of its low exposure and intuitive viewing of 3D structures, CBCT is widely used in hospitals these days. Since it is taken before and after surgery, during diagnosis, planning, and follow-up, it is possible to evaluate hard tissue and soft tissue in 3D for pharyngeal airway. There have been many studies on pharyngeal airway analysis through CT after mandible setback surgery, and various reference points, planes, and measurements were used [5, 12, 25]. In this study, the changes in pharyngeal airway after two types of mandibular setback surgery were investigated. The InVivo5 program was used for CT analysis, and since the measured values for the airway may vary depending on the posture, re-orientation was performed based on the right porion and the Frankfort horizontal plane passing through the orbitalia with the nasion as the origin. The landmarks, sella (Se), PNS, P, E, B point, were indicated, and Nph AP, Oph AP, and Hph AP were measured for AP distance, and Nph vol, Oph vol, Hph vol, and whole pharyngeal volume were measured to obtain volumetric change. The SV-B distance was measured to find out the amount of mandible setback.

Holmberg and Linder-Aronson [26] found that Nph is not affected by mandibular setback surgery. Wenzel et al. [27] reported that after BSSRO setback surgery, Nph AP decreased by 2.3 mm after surgery and maintained until 1 year. In the present study, there was no significant change in Nph vol after surgery in group 1 and group 2 patients, and this was the same as that of Hatab et al. [5], Uesugi et al. [12], and Park et al. [25]. Most of the studies reported that Nph measurements were not related to surgery in both group 1 and group 2. In our study, in group 1, the direction of posterior movement of the mandible and the Nph space were anatomically far apart, so it seems that there was no difference before and after surgery. In group 2 patients, Nph AP decreased at postoperative 6 months (T0–T2, *p* < 0.05) and then increased at 1 year after surgery (T2–T3, *p* < 0.01) and relapsed (T0–T3, *p* > 0.05).

Enacar et al. [28] found that the volume of the oropharyngeal airway remained reduced for more than 1 year and 6 months in mandibular setback surgery patients. The hyoid bone and lower tongue point moved down, and accordingly, the hypopharyngeal airway space was significantly reduced. Tselink and Pogrel [29] and Hochban et al. [30], however, found that the oropharyngeal airway reduced postoperatively and recovered. In the present study, oropharyngeal and hypopharyngeal volume significantly decreased and maintained for up to 1 year in group 1. Park et al. [25] and Greco et al. [31] suggested that the Oph was affected more by mandibular setback because it is the close to the mandible and the tongue anatomically. In this study, in group 2, Oph vol were decreased at 6 months postoperatively (T0–T2, *p* < 0.05) but increased and relapsed significantly at the T2–T3 period. There was no significant change in measurement at 1 year after surgery (T0–T3, *p* > 0.05). This is consistent with the results of previous papers that the reduction of airway diminished after surgery [13, 14, 25].

The change of whole pharynx volume was significantly decreased and maintained in group 1, but in group 2, there was a tendency to relapse after postoperative reduction, and no significant change was observed at 1 year postoperatively (T0–T3, *p* > 0.05).

When comparing between group 1 and group 2, the decrease in Oph vol and whole pharynx volume was significantly greater in group 1 up to 1 year after surgery (*p* < 0.01). In this study, the bimaxillary surgery group was accompanied by an average 4.23 mm of posterior impaction and 0.81 mm advance of the maxilla. This movement causes upward and forward movement of the posterior nasal spine, attached muscles, and soft tissues and it could reduce the effect of narrowing upper airway space, against the posterior movement of the mandible.

In the present study, no patients complained of postoperative OSA or severe and persistent snoring at 1 year postoperatively. However, after orthognathic surgery, especially when only mandibular setback was performed, the volume and distance of the pharyngeal airway were decreased, so a long-term follow-up is required for airway-related symptoms such as snoring and OSA.

## Conclusion

In class III malocclusion patients, mandibular setback surgery only showed a greater reduction in pharyngeal airway than bimaxillary surgery with posterior impaction, and bimaxillary surgery was more stable in terms of airway. Therefore, it is important to evaluate the airway before surgery and include it in the surgical plan.

## Data Availability

The datasets used and analyzed during the current study are available from the corresponding author on reasonable request.

## Abbreviations

BSSRO: Bilateral sagittal split ramus osteotomy
OSA: Obstructive sleep apnea
AP: Anteroposterior
3D: Three-dimensional
CBCT: Cone-beam computed tomography
CT: Computed tomography
DICOM: Digital imaging and communications in medicine
Nph: Nasopharynx
Oph: Oropharynx
Hph: Hypopharynx
SV-B: The distance from the sella vertical plane to the B point
Se: Sella
PNS: Posterior nasal spine
Vol: Volume
